# The Institutional Library

**Published:** 1917-10-06

**Authors:** 


### THE INSTITUTIONAL LIBRARY.

**Hygiene and Public Health.** By Dr. Louis C. Parkes and Dr. H. R. Kenwood. Sixth Edition. Demy 8vo. Pp. 787. (London: H. K. Lewis and Co., Ltd. 14s. net.)

To the student of medical literature it is a matter of interest, not devoid of an element of sadness, to watch the waxing and waning of text-books in popularity. With the best of them it seems that there comes, sooner or later, a time when the personal influence which made for success fails. In spite of (sometimes perhaps on account of) the introduction of new blood in editorship, a rot sets in; the book has had its day, and, like the patches in the old garment, the emendations and interpolations serve only to show how threadbare has become the original fabric of the composition. To the practitioner and student of medicine there can be but one set of text-books: those which belonged to his essentially student days, to his hospital-training epoch. As the years go by he turns from time to time to the yellow pages of his Erichsen or his old edition of Gray with a fondness but little marred by his realisation and regret that by modern standards the book is obsolete.

It is to be feared that from the purely literary standpoint there is a sad falling away in text-books, though this decline is by no means limited or peculiar to medical works. We seek in vain to-day for such graphic descriptive symptomatology as that of Bristowe's "Medicine," or for so elegant a production of author and publisher as Playfair's "Midwifery." The text-book has become more a compilation and less a holograph; the annotator, the précis-writer, and the shorthand-typist have set their mark upon it. The result is a book infinitely more informative, but less easy and pleasurable to read, because less attractive in style and in matter.

Parkes and Kenwood's "Hygiene and Public Health" is a text-book *par excellence* in the zenith of a reputation which shows no sign of failing. It was in 1889 that Messrs. H. K. Lewis published for Dr. Louis Parkes the excellent book which has developed into the present sixth edition under his conjoint authorship with Dr. Henry R. Kenwood. The volume has grown in bulk and weight to nearly double its original size, as grow it must to keep pace with the expanding scope of its subject and of the examinations for sanitary science diplomas. The text-book must follow the examiner just as the examiner is, in some degree, inspired by the text-book; and a students' book of hygiene must cover the ground—from glanders to relative humidity, from milk to soil-pipes, from birth-rates to burial. The book amply fulfils its function; not only has it lost nothing in its growth, but it has kept well in the front rank of standard medical works. Apart from careful revision and the addition of much fresh matter, this edition contains a new chapter on maternity and child-welfare work and a note on marine hygiene as applied to merchant and cattle ships: as a sign of the times the section on camp sanitation has been amplified.

Readers of The Hospital will understand why we turn to the chapters on the ventilation of hospitals and public buildings. The "Plenum" system is, we are told, "not now regarded with favour for hospital purposes." "Air which has travelled through lengthy shafts and special air chambers . . . is liable to cause lassitude and a feeling of depression" and "there is reason to believe that the vitalising principle characteristic of really fresh air is diminished." The ventilation in the House of Commons "does not appear to be very satisfactory, and is frequently complained of": members of Parliament have, we think, often used language on the subject considerably plainer than is here suggested! We find no reference to the question of the influence upon the oxygen-content of "washing," by sprays or wet screens, air which is being prepared for use in artificial "ventilation." It is a question of much importance, and one into which further inquiry is highly desirable.

**The Indian Operation of Couching for Cataract.** By Robert Henry Elliot, M.D., B.Sc. Lond., Sc.D. Edin., F.R.C.S., Lieut.-Colonel I.M.S. retired; late Superintendent of the Government Ophthalmic Hospital, Madras, etc. With forty-five illustrations. (London: H. K. Lewis and Co., Ltd. 1917. Pp. 94. Price 7s. 6d. net.)

The earliest attempts to remove the obstruction to vision caused by an opaque lens have been lost in the obscurity of the past; but when we first hear of it, it is already established. We find a very definite operation excellently described in the pages of Celsus, who probably lived in the first century of our present era, and Celsus himself appears to have derived his knowledge of the operation from the writings of Philoxenes, who lived more than three hundred years earlier. The operation employed at this early date in the history of surgery was in a way extremely simple, for it consisted in nothing more than displacing the opaque lens so that it was no longer in the path of vision. Although the operation was frequently unsuccessful, its results were sometimes satisfactory enough to induce surgeons to perform it, and for patients to submit to it during the long period of more than two thousand years. Here and there through the centuries we find accounts of the operation, all written along the lines of the operation described by Celsus, and though there are differences these are slight, for there was no essential change in the manner in which it was performed. Although nowadays extraction is the recognised operation in all civilised countries, and although extraction had been attempted even many centuries ago, it was not until the year 1745 that Daviel, a French surgeon, placed the operation of extraction on a reasonable basis. Yet practically another century had to pass by until extraction became the only operation employed by surgeons in Europe. Nevertheless in uncivilised countries, or semi-civilised countries such as India and China, the method of couching for cataract still holds sway amongst the native surgeons, who have not been trained in modern schools of medicine. In India at the present time couching is widely performed by native operators, whose knowledge is small indeed, and whose practice is based on traditional teaching. Their instruments are of the simplest nature, and always so septic that it is a wonder that any of their cases benefit in the slightest degree. It is not surprising that British surgeons practising in India should feel great interest in the results of the operation, and from their position they have been able to settle the question whether the operation of couching should ever be performed. This book by Colonel Elliot has been written to put on record what the operation is and what its results are. His opinion is based on 780 cases of couching performed by native surgeons. In only about 10 per cent. of the cases is there any real improvement of vision, and most of these were recent cases, and therefore not unlikely to lose later some of the benefit they had at first gained. In opposition to these dreadful results may be placed the statistics of the extraction operation at the Madras Hospital. Recoveries numbered 96 per cent., poor results formed 2 per cent., and failures 2 per cent. The cases operated on by couching also show that in not a few instances the native operator mistook for cataract cases of blindness arising from other causes, and therefore these were cases in which the operation could not possibly do any good. The whole book shows how carefully and judiciously Colonel Elliot has examined into this important subject. The most real conclusion that is to be drawn from the book is the immense need existing for the further extension in India of European medical science. We are sure that the Government of India is doing much to carry to the ignorant natives the blessings of Western science, but much more remains to be done. The harvest truly is plenteous, but the labourers are few.

**Food and Fitness; or, Diet in Relation to Health.** By James Long. (London: Chapman and Hall, Ltd. 1917. 5s. net.)

"If alcohol kills its thousands, excessive eating and the consumption of improper food kill their thousands too," says Mr. James Long, and further declares that "science has determined with some accuracy not only what foods, but what quantities of those foods, domestic animals require, both under conditions of labour and inactivity, for the maintenance of their health and productive power. Man ignores all teaching in the same direction, and, having eaten enough for two or three people, or eaten unsuitable foods and been attacked by some disease in consequence, he prays for recovery from the trouble which he says God has 'inflicted' on him. The disease is not the work of a merciful Creator; it is self-inflicted; it is the harvest which follows the sowing of the seed."

This is in epitome the burden of Mr. Long's book, and he enforces the lesson in language so clear and simple that all can understand, and so ably illustrates the teaching of science by practical recipes that to eat less and live well within the limitations imposed by the Food Controller becomes a pleasure and not a martyrdom. After explaining with lucidity the component parts of food, the food value of proteids, carbo-hydrates, fats, and mineral salts, and the meaning of calories, he proceeds to estimate these by pennyworths; thus, if bread is 2d. a pound, a penny will buy 800 units or calories; beef, at 10d. a pound, will furnish only 85 calories for a penny. The comparative value of a meat or meatless dinner for a family consisting of a man, his wife, and three children is clearly illustrated by tables, the number of calories in each item of the menu and the cost being given. Our author is not, however, a professed vegetarian, and gives some tasty recipes in which meat plays a part, but he is convinced that the practice of eating meat daily, or two or three times a day, is a dangerous and disease-producing habit to those who lead sedentary lives or who are over forty years of age. In the chapters on "The Most Economical Foodstuffs" valuable information is given, and the advantages of whole-meal bread, the economy of home-made bread, the food value of skim milk, and the use of oatmeal, "the best balanced food of all the cereals," are described in detail. The chapters on "Vegetables and Fruit as Foods" cannot fail to interest the housekeeper, containing as they do many recipes for cooking vegetables alone or with meat. Of the nutritive value of fruit much is told, and we learn that when apples cost 2d. a pound one penny will buy 100 units of energy, and dates at 3d. a pound will provide 500 units for a penny. Attention is also drawn to the neglected food of our hedgerows and commons, to blackberries, and whortleberries, and we are assured that an excellent porridge can be made of whortleberries passed through a mincing-machine and mixed with soaked prepared oats, or ground nuts, and condensed milk. Now is the time to take to heart the lessons taught by Mr. James Long, and to learn by practical experience how life can be maintained, health improved, and energy increased by consuming less food, and food of the best nutritive value. We heartily commend "Food and Fitness" as a book of marked value to the public, especially at the present time, when to know how to be well fed economically is one of the means of winning the war.

**The King's Fishing.** Done into Verse by Charles Mercier, M.C.C. (London: The Mental Culture Enterprise, 329 High Holborn, W.C. 1. Price 1s. 3d. net.)

This volume consists of a short collection of parodies, followed by a number of critical and explanatory notes, which satirise the methods and style of the commentators. It takes a lover of literature to write a good parody, and therefore we presume that Dr. Mercier's favourite and most admired authors are those after whose manner his verses are composed: Coleridge, Scott, Dr. Johnson, and if not Spenser, at all events writers who affect the Spenserean stanza. The best seems to us to be "The Ballad of the King's Fishing," which is a neat parody of "The Rhyme of the Ancient Mariner." The running argument in the margin is especially successful. Then we come to "The Vanity of River Fishes," in imitation of Dr. Johnson, in which the phrase "pluvious moisture" (for rain) occurs. Dr. Johnson was his own best parodist, as great stylists, in fact, generally are; while in a similar vein of exuberant diction Dr. Erasmus Darwin has never been surpassed. His "Botanic Garden" and "The Loves of the Plants" were so incredible in style that it was impossible to exceed their unconscious humour until a writer appeared in Frere, who wrote lines which are not to be distinguished from their originals. Some of these were quoted in The Hospital in an article on Dr. Erasmus Darwin, which appeared on March 4, 1916. But this, the fine flower of parody, is very rare. Dr. Mercier, as a distinguished *dilettante* of letters, seeks only, we suspect, to amuse his leisure and our own; but the quality of his book, and consequently his discrimination as a critic of literature, are seen in the absence of any attempt to overreach himself. There are no lapses from parody to piffle. The notes, too, are written in a manner to delight the student of Theobald, and of the run of Shakespearean and classical commentators. How much he must have chuckled, half indulgently and half petulantly, over the originals! Dr. Mercier, the enemy of pedants, yet has a prejudice or two of his own. He refuses, as a purist, to allow that a disease may "develop," and in the volume before us he declares that the word "meticulous" is "beloved of the precious and loathed by every English scholar." It may be loved by the precious, but it is an honest word to which there is no valid objection except in the writings of those who make almost any word objectionable. In its primary meaning (timid), it was good enough for Sir Thomas Browne. In its secondary sense—namely, over-scrupulous—it was used by J. A. Symonds, a painstaking, though far from first-class, writer. Probably better examples exist, as well as worse ones. Why is it not good enough for Dr. Mercier? We mention his disapproval to show that he is himself a purist; indeed in his use of words he is meticulous to a degree of needless scrupulosity! This quality in his criticism makes "The King's Fishing" a delightful book for all who have browsed in the fields of English literature.

